# Sphingosine 1‐phosphate receptor modulators in multiple sclerosis treatment: A practical review

**DOI:** 10.1002/acn3.52017

**Published:** 2024-02-16

**Authors:** Patricia K. Coyle, Mark S. Freedman, Bruce A. Cohen, Bruce A. C. Cree, Clyde E. Markowitz

**Affiliations:** ^1^ Department of Neurology, Stony Brook Renaissance School of Medicine Stony Brook University Stony Brook New York USA; ^2^ University of Ottawa Department of Medicine and the Ottawa Hospital Research Institute Ottawa Ontario Canada; ^3^ Department of Neurology Northwestern University, Feinberg School of Medicine Chicago Illinois USA; ^4^ Weill Institute for Neurosciences, Department of Neurology University of California San Francisco San Francisco California USA; ^5^ Department of Neurology, Perelman School of Medicine University of Pennsylvania Philadelphia Pennsylvania USA

## Abstract

Four sphingosine 1‐phosphate (S1P) receptor modulators (fingolimod, ozanimod, ponesimod, and siponimod) are approved by the US Food and Drug Administration for the treatment of multiple sclerosis. This review summarizes efficacy and safety data on these S1P receptor modulators, with an emphasis on similarities and differences. Efficacy data from the pivotal clinical trials are generally similar for the four agents. However, because no head‐to‐head clinical studies were conducted, direct efficacy comparisons cannot be made. Based on the adverse event profile of S1P receptor modulators, continued and regular monitoring of patients during treatment will be instructive. Notably, the authors recommend paying attention to the cardiac monitoring guidelines for these drugs, and when indicated screening for macular edema and cutaneous malignancies before starting treatment. To obtain the best outcome, clinicians should choose the drug based on disease type, history, and concomitant medications for each patient. Real‐world data should help to determine whether there are meaningful differences in efficacy or side effects between these agents.

## Background

Sphingosine 1‐phosphate (S1P) receptor modulators are G protein‐coupled receptors found throughout the body. They mediate a broad range of functions via five distinct subtypes (S1P_1–5_).^1^, ^2^ Owing to the widespread expression of these receptor subtypes across multiple organ systems, they can influence the immune system, brain, lung, liver, heart, and vasculature (Fig. S1).^1^, ^2^ Considering the extensive presence of S1P receptors on cardiomyocytes and vascular endothelial cells, S1P receptor modulators are expected to have cardiovascular effects.^3^ These were observed in preclinical studies, particularly for S1P receptor modulators that target S1P_1_ and S1P_3_.^4^

Owing to their ability to bind with high affinity to one or more S1P receptor subtypes, S1P receptor modulators have a complex mechanism of action with both peripheral immunological and central nervous system (CNS) effects.^1^, ^5^ Consequently, S1P receptor modulators represent a therapeutic option for immune‐mediated diseases, including multiple sclerosis (MS).^1^, ^2^ To date, four S1P receptor modulators, fingolimod, ozanimod, ponesimod, and siponimod, are approved by the US Food and Drug Administration (FDA) and the European Medicines Agency (EMA) for the treatment of MS (Table 1).^6^, ^7^, ^8^, ^9^, ^10^, ^11^, ^12^, ^13^ S1P receptor modulators function by blocking the capacity of lymphocytes to egress from lymph nodes, leading to a reduction in the number of lymphocytes in peripheral blood.^14^ While the full mechanistic pathway by which S1P receptor modulators exert therapeutic effects is unknown, lymphocyte sequestration and reduction of lymphocyte migration into the CNS were proposed as the primary mechanism of action in MS.^6^, ^7^, ^8^, ^9^

**Table 1:** Indications and dosing of the different S1P receptor modulators.

	Fingolimod^6^ S1P_1_, S1P_3_, S1P_4_, and S1P_5_	Ozanimod^7^ S1P_1_ and S1P_5_	Ponesimod^8^ S1P_1_	Siponimod^9^ S1P_1_ and S1P_5_
Indication(s)	Relapsing forms of MS, including CIS, RRMS, and active SPMS, in patients ≥10 years of age	Relapsing forms of MS, including CIS, RRMS, and active SPMS, in adults Moderately to severely active UC in adults	Relapsing forms of MS, including CIS, RRMS, and active SPMS, in adults	Relapsing forms of MS, including CIS, RRMS, and active SPMS, in adults
Recommended dosing	For adults and pediatric patients ≥10 years of age weighing >40 kg, 0.5 mg orally QD For pediatric patients ≥10 years weighing ≤40 kg, 0.25 mg orally QD	After initial titration, 0.92 mg orally QD	After initial titration, 20 mg orally QD	After initial titration, 2 mg orally QD Dosage adjustment to 1 mg QD is required in patients with a CYP2C9*1/*3 or *2/*3 genotype
Pharmacokinetics				
T_max_	12–16 h	6–8 h CC112273 and CC1084037 (active metabolites): 10 and 16 h	2–4 h	3–8 h
Half‐life	6–9 days	Ozanimod: 21 h CC112273 and CC1084037 (active metabolites): 10 days	33 h	~30 h
Time to reach steady state	1–2 months	Ozanimod: 102 h CC112273 (major active metabolite): 45 days	3 days of maintenance dose	6 days
Contraindications	In the last 6 months experienced myocardial infarction, unstable angina, stroke, TIA, decompensated heart failure requiring hospitalization or Class IIIor IV heart failure History or presence of Mobitz type II second‐degree or third‐degree AV block or sick sinus syndrome, unless patient has a functioning pacemaker A baseline QTc interval ≥ 500 msec Cardiac arrhythmias requiring anti‐arrhythmic treatment with Class Ia or III anti‐arrhythmic drugs Had a hypersensitivity reaction to fingolimod or any of the excipients in fingolimod. Observed reactions include rash, urticaria, and angioedema upon treatment initiation	In the last 6 months, experienced a myocardial infarction, unstable angina, stroke, TIA, decompensated heart failure requiring hospitalization, or Class III or IV heart failure Presence of Mobitz type II second‐degree or third‐degree AV block, sick sinus syndrome, or sino‐atrial block, unless the patient has a functioning pacemaker Severe untreated sleep apnea Taking an MAO inhibitor	In the last 6 months, experienced myocardial infarction, unstable angina, stroke, TIA, decompensated heart failure requiring hospitalization, or Class Ill or IV heart failure Presence of Mobitz type II second‐degree, third‐degree AV block, sick sinus syndrome, or sino‐atrial block, unless the patient has a functioning pacemaker	A CYP2C9*3/*3 genotype In the last 6 months experienced myocardial infarction, unstable angina, stroke, TIA, decompensated heart failure requiring hospitalization, or Class III or IV heart failure Presence of Mobitz type II second‐degree, third‐degree AV block, or sick sinus syndrome, unless patient has a functioning pacemaker

AV, atrioventricular; CIS, clinically isolated syndrome; h, hour; MAO, monoamine oxidase; MS, multiple sclerosis; QD, once daily; RRMS, relapsing–remitting multiple sclerosis; S1P_1_, sphingosine 1‐phosphate receptor subtype 1; S1P_3_, sphingosine 1‐phosphate receptor subtype 3; S1P_4_, sphingosine 1‐phosphate receptor subtype 4; S1P_5_, sphingosine 1‐phosphate receptor subtype 5; SPMS, secondary progressive MS; T_max_ time of peak plasma concentration; TIA; transient ischemic attack; UC, ulcerative colitis.

Fingolimod, a nonselective S1P receptor modulator targeting S1P_1_, S1P_3_, S1P_4_, and S1P_5_, was approved in the US in 2010 and the EU in 2011 as the first oral capsule for the treatment of MS.^10^, ^15^, ^16^ Fingolimod, the only S1P receptor modulator studied in children with MS, is indicated for patients at least 10 years of age with relapsing MS (RMS), including clinically isolated syndrome (CIS, US only), relapsing–remitting MS (RRMS), and active secondary progressive MS (SPMS).^6^ Fingolimod is a prodrug that requires phosphorylation to be converted to its active form, which is thought to contribute to its relatively long elimination half‐life.^2^ Safety concerns, particularly the cardiac‐related effects (e.g., bradycardia) associated with fingolimod led to the development of more selective, second‐generation S1P receptor modulators: ozanimod, ponesimod, and siponimod.^17^ The FDA recently approved an orally disintegrating fingolimod tablet for the treatment of adult patients with RMS, which provides faster onset and improved bioavailability than the oral capsule formulation.^18^

The second generation of S1P receptor modulators do not require phosphorylation for activity and also have shorter half‐lives, implying a shorter washout period.^15^ The exception is ozanimod, which has active metabolites with a longer mean elimination half‐life than fingolimod (Table 1).^19^ Siponimod and ozanimod, which are selective for S1P_1_ and S1P_5_, received FDA approval in 2019 and 2020, respectively, for the treatment of adults with RMS, including CIS, RRMS, and active SPMS.^7^, ^9^ The EMA approved siponimod in 2020 for the treatment of SPMS and ozanimod in 2020 for the treatment of active RRMS.^11^, ^12^ Additionally, in 2021, ozanimod was the first in this class to receive FDA approval for moderately to severely active ulcerative colitis.^7^, ^20^ Ponesimod is selective for S1P_1_, and received FDA approval in 2021 for the treatment of adults with RMS, including CIS, RRMS, and active SPMS, and EMA approval in 2021 for the treatment of adults with active RMS.^8^, ^13^

S1P receptor modulators are of particular interest in the MS treatment landscape because they may have direct CNS effects and are able to cross the blood–brain barrier, whereas several other disease‐modifying therapies (DMTs) (e.g., interferons, glatiramer acetate, and monoclonal antibodies) function only in the periphery.^21^, ^22^ A direct CNS effect, which is supported by preclinical evidence, may contribute to clinical benefits in MS, because S1P receptors are expressed by cells in the CNS.^17^ This direct mechanism of action potentially provides a benefit for S1P receptor modulators in the treatment of MS; however, clinical studies have not substantiated these findings. Notably, clinical studies clearly demonstrated reductions in brain volume loss, as is common with DMTs and particularly at early stages of disease,^23^ suggesting use of these agents early in the course of MS rather than waiting until progression is clinically evident.^24^

Considering the plethora of recent and ongoing research on S1P receptor modulators, this review aimed to summarize the data generated to date on the efficacy and safety of S1P receptor modulators for the treatment of MS, with an emphasis on detailing the similarities and differences of each agent.

## Efficacy of S1P Receptor Modulators in Multiple Sclerosis

The efficacies of fingolimod, ozanimod, ponesimod, and siponimod were evaluated in phase 2 and phase 3 clinical trials. Efficacy data from the pivotal phase three trials for the key endpoints listed in the respective prescribing information for each drug are summarized in Table 2. The safety and efficacy of fingolimod was assessed in adults with RRMS (FREEDOMS,^25^ FREEDOMS II,^30^ and TRANSFORMS^31^) and in pediatric participants with RMS (PARADIGMS^32^); siponimod in adults with SPMS (EXPAND^29^); ozanimod in adults with RMS (SUNBEAM^26^ and RADIANCE^27^); and ponesimod in adults with RMS (OPTIMUM).^28^ The safety and efficacy of fingolimod has also been assessed in adults with primary progressive MS (INFORMS).^33^ However, in this phase 3 trial, fingolimod did not slow disease progression compared with placebo,^33^ suggesting that CNS penetration of fingolimod alone was inadequate to alter the course of progression in primary progressive MS. The results of this study are not further discussed in this review.

**Table 2:** Overview of efficacy data from adult MS registrational trials of the different S1P receptor modulators.

	Fingolimod^6^, ^25^ S1P_1_, S1P_3_, S1P_4_, and S1P_5_	Ozanimod^7^, ^26^, ^27^ S1P_1_ and S1P_5_	Ponesimod^8^, ^28^ S1P_1_	Siponimod^9^, ^29^ S1P_1_ and S1P_5_
Comparator	PBO (FREEDOMS, 2 years) IFN β‐1a (TRANSFORMS, 1 year)	IFN β‐1a (SUNBEAM, ≥ 1 year; RADIANCE, 2 years)	Teriflunomide (OPTIMUM, 108 weeks)	PBO (EXPAND, 21‐month median study duration)
Relapse				
ARR	0.18 vs. 0.40 (PBO), *p* < 0.001 0.16 vs. 0.33 (IFN β‐1a), *p* < 0.001	0.18 vs. 0.35 (IFN β‐1a); relative reduction 48%, *p* < 0.0001 0.17 vs. 0.28 (IFN β‐1a); relative reduction 38%, *p* < 0.0001	0.20 vs. 0.29 (teriflunomide); relative reduction 31%, *p* = 0.0003	0.07 vs. 0.16 (PBO); relative reduction 55%, *p* < 0.01
Percentage of patients without relapse	70% vs. 46% (PBO), *p* < 0.001 83% vs. 70% (IFN β‐1a), *p* < 0.001	78% vs. 66% (IFN β‐1a) 76% vs. 64% (IFN β‐1a)	71% vs. 61% (teriflunomide)	89% vs. 81% (PBO)
Confirmed disability progression (CDP)				
3‐month CDP	18% vs. 24% (PBO), *p* = 0.02	8% vs. 8% (IFN β‐1a), NS (SUNBEAM and RADIANCE combined)	11% vs. 13% (teriflunomide), NS	26% vs. 32% (PBO), *p* = 0.013
3‐month CDP hazard ratio	0.70 (95% CI 0.52, 0.96), *p* = 0.02 0.71 (95% CI 0.42, 1.21), *p* = 0.21	0.95 (*p* = 0.77) (SUNBEAM and RADIANCE combined)	0.83, *p* = 0.29	0.79 (95% CI 0.65, 0.95), *p* = 0.013
MRI outcomes				
Mean (median) number of GdE lesions	0.2 (0) vs. 1.1 (0) (PBO), *p* < 0.001 0.2 (0) vs. 0.5 (0) (IFN β‐1a), *p* < 0.001	0.2 vs. 0.4 (IFN β‐1a); relative reduction 63%, *p* < 0.0001 0.2 vs. 0.4 (IFN β‐1a); relative reduction 53%, *p* = 0.0006	0.2 vs. 0.4 (teriflunomide); relative reduction 59%, *p* < 0.0001	0.1 vs. 0.6 (PBO); relative reduction 86%, *p* < 0.0001
Mean number of new/enlarging T2 lesions	2.5 (0) vs. 9.8 (5.0) (PBO), *p* < 0.001 1.6 (0) vs. 2.6 (1.0) (IFN β‐1a), *p* = 0.002	1.5 vs. 2.8 (IFN β‐1a); relative reduction 48%, *p* < 0.0001 1.8 vs. 3.2 (IFN β‐1a); relative reduction 42%, *p* < 0.0001	1.4 vs. 3.2 (teriflunomide); relative reduction 56%, *p* < 0.0001	0.7 vs. 3.6 (PBO); relative reduction 81%, *p* < 0.0001

ARR, annualized relapse rate; CDP, confirmed disability progression; CI, confidence interval; GdE, gadolinium‐enhancing; IFN, interferon; MRI, magnetic resonance imaging; MS, multiple sclerosis; NS, not significant; PBO, placebo; S1P_1_, sphingosine 1‐phosphate receptor subtype 1; S1P_3_, sphingosine 1‐phosphate receptor subtype 3; S1P_4_, sphingosine 1‐phosphate receptor subtype 4; S1P_5_, sphingosine 1‐phosphate receptor subtype 5.

In the pivotal phase 3 trials, treatment with fingolimod^25^ or siponimod^29^ resulted in statistically significantly lower annualized relapse rates (ARR) compared with placebo, while fingolimod,^31^ ozanimod,^26^, ^27^ and ponesimod^28^ showed statistically significantly lower ARR compared with active comparators. The proportion of patients without relapse was higher with fingolimod compared with placebo and intramuscular interferon beta‐1a (IFN β‐1a),^25^, ^31^ ozanimod compared with IFN β‐1a,^26^, ^27^ ponesimod compared with teriflunomide,^28^ and siponimod compared with placebo.^29^ Treatment with fingolimod^25^ and siponimod^29^ resulted in a significantly lower proportion of patients with 3‐month confirmed disability progression (CDP) compared with placebo. The mean numbers of gadolinium‐enhancing and new/enlarging T2 lesions were significantly reduced with fingolimod compared with placebo and IFN β‐1a,^25^, ^31^ ozanimod compared with IFN β‐1a,^26^, ^27^ ponesimod relative to teriflunomide,^28^ and siponimod compared with placebo.^29^

In terms of durability of efficacy, the response to S1P receptor modulator treatment appears to be sustained long term. In LONGTERMS, an open‐label, phase 3b extension study, sustained efficacy was reported with fingolimod over 10 years in more than 4000 participants with MS who had participated in the core trials.^34^ Similarly, data from the open‐label extension DAYBREAK study, ponesimod phase 2 extension study, and phase 3 EXPAND study confirmed maintenance of efficacy of ozanimod for up to 8 continuous years of exposure, ponesimod for up to 8 years, and siponimod for more than 5 years, respectively.^35^, ^36^, ^37^

Overall, while the efficacy data between the pivotal clinical trials of fingolimod, ozanimod, ponesimod, and siponimod are generally comparable, head‐to‐head clinical trials were not conducted; therefore, definitive conclusions comparing the efficacy of S1P receptor modulators cannot be made.^2^

## Safety of S1P Receptor Modulators in Multiple Sclerosis

There are no direct comparative trials between S1P receptor modulators, and the currently available safety information for agents is from their individual trials; this should be considered when comparing event rates between agents.

### Adverse events

The varying effects of S1P receptor modulators on different receptor subtypes (S1P_1_, S1P_3_, S1P_4_, and S1P_5_) may increase the risk of certain AEs, because these receptors are expressed on cells throughout the body.^15^ Therefore, it is important for clinicians to consider specific events known to be associated with a particular S1P receptor modulator when considering a treatment approach. Notably, pretreatment screening and continued follow‐up can minimize risks, with the overall safety profile of this drug class remaining favorable.^38^ The most relevant AEs encountered with S1P receptor modulators include cardiovascular AEs, lymphopenia, serious infections, malignancy (including cutaneous malignancies), hepatotoxicity, seizures (mainly in children), and pulmonary effects (Table 3).^42^

**Table 3:** Overview of safety data of the different S1P receptor modulators.

	Fingolimod^6^, ^25^, ^39^ S1P_1_, S1P_3_, S1P_4_, and S1P_5_	Ozanimod^7^, ^26^, ^27^, ^40^ S1P_1_ and S1P_5_	Ponesimod^8^, ^28^ S1P_1_	Siponimod^9^, ^29^ S1P_1_ and S1P_5_
Cardiac and vascular				
HR reduction from baseline	8–10 bpm	1.2 bpm	6 bpm	5–6 bpm
HR < 40 bpm	Rare	Not observed	Rare (3 patients)	Rare
Bradycardia	0.6% vs. 0.1% (PBO) symptomatic following first dose (hypotension, dizziness, fatigue, palpitations, and/or chest pain that usually resolved within the first 24 h on treatment)	First dose: 0.6% vs. 0.0% (IFN β‐1a) After day 1: 0.8% vs. 0.7% (IFN β‐1a)	Treatment initiation: 5.8% vs. 1.6% (teriflunomide)	4.4% vs. 2.9% (PBO); generally asymptomatic; symptomatic patients reported dizziness or fatigue that resolved within 24 h without treatment
AV blocks	Conduction abnormalities were generally transient, asymptomatic, and resolved within the first 24 h	There were no cases of Mobitz type 2 second‐ or third‐degree AV blocks in patients who underwent dose titration	Conduction abnormalities were generally transient, asymptomatic, and resolved within 24 h	Conduction abnormalities were generally transient, asymptomatic, and resolved within 24 h
Patients with first‐degree AV block	4.7% vs. 1.6% (PBO)	0.6% vs. 0.2% (IFN β‐1a)	3.4% vs. 1.2% (teriflunomide)	5.1% vs. 1.9% (PBO)
Patients with second‐degree AV block or higher	Second‐degree: 4% vs. 2% (PBO) Third‐degree occurred in postmarketing	No report	No report	<1.7% (second‐degree)
QTc prolongation	14.0 msec (1.25 and 2.5 mg doses)	No report	11.8 msec (40 mg)	7.8 msec (2 mg dose)
Hypertension	8% vs. 4% (PBO); Compared with PBO, patients treated with fingolimod 0.5 mg had a mean increase in SBP of 3 mm Hg and in DBP of 2 mm Hg developing after 1 month of treatment and persisting with continued treatment	3.9% vs. 2.1% (IFN β‐1a); Compared with IFN β‐1a, ozanimod treatment led to an increase of 1–2 mm Hg in SBP, with no change in DBP, developing after 3 months of treatment and persisting with continued treatment	10.1% vs. 9.0% (teriflunomide); Ponesimod increased SBP by a mean of 2.9 mm Hg and DBP by 2.8 mm Hg (compared with 2.8 mm Hg and 3.1 mm Hg in teriflunomide, respectively). Increases were first seen after 1 month of treatment and persisting with continued treatment	12.5% vs. 9.2% (PBO); Compared with PBO, patients treated with siponimod had an average increase in SBP of 3 mm Hg and DBP of 1.2 mm Hg developing after 1 month of treatment and persisting with continued treatment
Macular edema	0.5% (0.5 mg) vs. 0.4% (PBO)	0.3% vs. 0.3% (IFN β‐1a)	1.1% vs. 0% (teriflunomide)	1.8% vs. 0.2% (PBO)
Lymphopenia				
Lymphocyte reduction from baseline	70%	57%	60%–70%	70%–80%
Approximate ALC nadir	500 cells/μL	759 cells/μL	650 cells/μL	560 cells/μL
Lymphocyte recovery to normal range	1–2 months	30 days	1–2 weeks	10 days
Infections				
Common infections	Bronchitis, herpes zoster, influenza, sinusitis, and pneumonia were more common with fingolimod	Ozanimod increased the risk of viral upper respiratory tract infections, urinary tract infections, and herpes infections	Ponesimod increased the risk of upper respiratory tract infections; Similar rates of herpes zoster were reported in ponesimod and teriflunomide (4.8%)	Herpes zoster, herpes infections, bronchitis, sinusitis, upper respiratory infection, and fungal skin infection were more common with siponimod
Overall infections	72% (similar to PBO)	35% vs. 34% (IFN β‐1a)	54.2% vs. 52.1% (teriflunomide)	49.0% vs. 49.1% (PBO)
Herpetic infections	9% (0.5 mg) vs. 7% (PBO)	0.6% vs. 0.2% (IFN β‐1a) (only includes herpes zoster)	4.8% vs. 4.8% (teriflunomide)	4.6% vs. 3.0% (PBO)
Serious infections	2.3% (0.5 mg) vs. 1.6% (PBO)	1% vs. 0.8% (IFN β‐1a)	1.6% vs. 0.9% (teriflunomide)	2.9% vs. 2.5% (PBO)
Progressive multifocal leukoencephalopathy (PML)^a^	61 PML cases Incidence rate: 5.88 per 100,000 PY (August 2022)	1 PML case ~40,000 PY of exposure	No cases reported	3 PML cases ~30,000 PY of exposure (2022)
Cryptococcal meningitis (CM) infections	74 CM cases (9 per 100,000 PY)	No cases reported	No cases reported	1 CM case (>7236 PY)
Malignancies	Basal cell carcinoma rate: 2% vs. 1% (PBO) (FREEDOMS trials) Postmarket reports of melanoma, squamous cell carcinoma, Merkel cell carcinoma, and cases of lymphoma (T cell, B cell, CNS)	Melanoma, basal cell carcinoma, breast cancer, seminoma, cervical carcinoma, and adenocarcinomas, including rectal adenocarcinoma, were reported in controlled trials of MS and UC	Incidence of basal cell carcinoma was 0.4% (ponesimod) vs. 0.2% (teriflunomide); cases of other cutaneous malignancies, including melanoma, have been reported	Increased risk of basal cell (1.1%) and squamous cell carcinoma (0.2%) with siponimod treatment (EXPAND); cases of other cutaneous malignancies have been reported
Hepatic				
Hepatotoxicity	Clinically significant liver injury has occurred in the postmarket setting; cases of acute liver failure requiring liver transplant have been reported Levels of serum transaminase returned to normal within ~2 months following discontinuation	79% of patients with ALT elevations continued ozanimod treatment, with values decreasing within 2–4 weeks	The median time to 3x ULN of ALT was 3 months; the majority (89%) continued treatment and levels decreased within 2–4 weeks	Elevations in transaminases and bilirubin were reported in 10.1% (siponimod) vs. 3.7% (PBO). ALT returned to normal ~1 month after discontinuation
ALT ≥3 × ULN	14% vs. 3% (PBO)	5.5% vs. 3.1% (IFN β‐1a)	17.3% vs. 8.3% (teriflunomide)	5.6% vs. 1.5% (PBO)
ALT ≥5 × ULN	4.5% vs. 1% (PBO); most within 6–9 months of treatment initiation	1.6% vs. 1.3% (IFN β‐1a)	4.6% vs. 2.5% (teriflunomide)	1.4% vs. 0.5% (PBO); the majority occurred within 6 months of treatment initiation
Pulmonary	Reductions in FEV1 and DLCO The decrease from baseline in the percent of predicted values for FEV1 was 2.8% with fingolimod 0.5 mg and 1.0% for PBO; for DLCO, 3.3% and 0.5%, respectively Changes in FEV1 appear to be reversible after treatment discontinuation	Dose‐dependent reductions in FEV1 3 months after start of treatment In a pooled analysis, decline from baseline with ozanimod compared to IFN β‐1a was 60 mL (95% CI: −100, −20) at 12 months (mean difference 1.9%) Not enough evidence to determine reversibility of this effect	Dose‐dependent reductions in FEV1 and DLCO were observed mostly in the first month of treatment In OPTIMUM, the decrease from baseline in FEV1 at 2 years was 8.3% vs. 4.4% (teriflunomide)	Dose‐dependent reductions in absolute FEV1 after 3 months of treatment Decline in absolute FEV1 from baseline with siponimod vs. placebo was 88 mL at 2 years (mean difference, 2.8%) There is not enough evidence to determine the reversibility Changes in patients with MS who also had mild to moderate asthma and chronic obstructive pulmonary disease were similar to the overall population
Neurological				
Seizure^b^	0.9% (0.5 mg) vs. 0.3% (PBO)	NA	1.4% vs. 0.2% (teriflunomide)	1.7% vs. 0.4% (PBO)
AEs leading to discontinuation	FREEDOMS: 7.5% (fingolimod 0.5 mg) vs. 7.7% (PBO)	RADIANCE: 3.0% (1 mg) vs. 4.1% (IFN β‐1a) SUNBEAM: 2.9% (1 mg) vs. 3.6% (IFN β‐1a)	8.7% vs. 6.0% (teriflunomide)	8.5% vs. 5.1% (PBO)

AE, adverse event; ALC, absolute lymphocyte count; ALT, alanine transaminase; AV, atrioventricular; bpm, beats per minute; CM, cryptococcal meningitis; CNS, central nervous system; DPB, diastolic blood pressure; DLCO, diffusion lung capacity for carbon monoxide; FEV1, forced expiratory volume; h, hour; HR, heart rate; MS, multiple sclerosis; NA, not available; PBO, placebo; PML, progressive multifocal leukoencephalopathy; PY, person‐years; UC, ulcerative colitis; VZV, varicella zoster virus; QTc, corrected QT interval; ULN, upper limit of normal; S1P_1_, sphingosine 1‐phosphate receptor subtype 1; S1P_3_, sphingosine 1‐phosphate receptor subtype 3; S1P_4_, sphingosine 1‐phosphate receptor subtype 4; S1P_5_, sphingosine 1‐phosphate receptor subtype 5; SBP, systolic blood pressure.

^a^ PML is an opportunistic viral infection of the brain caused by the JC virus that typically occurs in patients who are immunocompromised and that usually leads to death or severe disability.

^b^ Mainly in children.^41^

Each S1P receptor modulator is associated with a transient reduction in heart rate upon initiation, that is highest with fingolimod (decrease of 8.1 beats per minute [bpm]), followed by ponesimod (decrease of 6 bpm), siponimod (decrease of 5–6 bpm), and ozanimod (decrease of 1.2 bpm).^6^, ^7^, ^8^, ^9^, ^43^ Meta‐analysis of S1P receptor modulator clinical trials showed that cardiovascular AEs occurred in 10.9% in patients treated with S1P receptor modulators compared with 4.8% in those receiving the control treatment, with a significantly increased risk of arrhythmia (primarily bradyarrhythmia) and hypertension in patients treated with S1P receptor modulators compared with control treatment.^3^ Fingolimod and siponimod are associated with greater rates of first‐degree AV block than placebo,^6^, ^9^ whereas ozanimod and ponesimod are associated with greater rates of bradycardia and first‐degree AV block than IFN β‐1a or teriflunomide, respectively.^7^, ^8^ Second‐ or third‐degree AV block was reported with fingolimod and siponimod^6^, ^9^ but not with ozanimod and ponesimod.^7^, ^8^ Fingolimod, ponesimod, and siponimod are associated with prolongation of QTc intervals,^6^, ^8^, ^9^ whereas ozanimod is not.^7^

While S1P receptor modulators were associated with an increased risk of macular edema,^42^ there was no increase in these events with ozanimod compared with IFN β‐1a in pivotal trials.^26^, ^27^ Macular edema was reported in 0.3% of patients treated with ozanimod and 0.3% of patients treated with IFN β‐1a.^7^ Fingolimod increases the risk of macular edema in a dose‐dependent manner,^6^ and in clinical trials, macular edema generally occurred in the first 3–4 months of treatment and partially or completely resolved after fingolimod discontinuation.^42^ Macular edema was reported in 1.1% and 1.8% of patients treated with ponesimod and siponimod, respectively, and in 0% and 0.2% of patients receiving teriflunomide or placebo, respectively.^8^, ^9^ Notably, patients with a history of uveitis, diabetes mellitus, and cataract surgery are at increased risk of macular edema during treatment with an S1P receptor modulator.^42^, ^44^

S1P receptor modulators reduce the capacity of lymphocytes to egress from lymph nodes, thereby reducing the number of lymphocytes in peripheral blood, which in turn can lower the ability of the immune system to fight infections.^2^, ^45^ Reductions in lymphocyte counts from baseline ranged from 57% with ozanimod to ~70% with fingolimod, siponimod, and ponesimod, with a nadir of ~760 cells/μL with ozanimod, 500 cells/μL with fingolimod, 560 cells/μL with siponimod, and 650 cells/μL with ponesimod (Table 3).^6^, ^8^, ^9^, ^26^, ^27^ Fastest lymphocyte recovery to normal range was 10 days with siponimod and 1–2 weeks with ponesimod, while it was 1 month with ozanimod and 1–2 months with fingolimod (Table 3).^6^, ^7^, ^8^, ^9^ All four S1P receptor modulators are associated with a risk of serious infections. In fingolimod trials, two patients died from herpetic infections, one due to disseminated primary herpes zoster and one to herpes simplex encephalitis. Both patients received high‐dose corticosteroids to treat suspected MS relapses.^6^ Other serious infections that occurred during S1P receptor modulator treatment include progressive multifocal leukoencephalopathy and cryptococcal meningitis (Table 3).

Basal cell carcinoma and other skin malignancies were reported as AEs for all four S1P receptor modulators.^6^, ^7^, ^8^, ^9^ Lymphoma, particularly non‐Hodgkin's lymphoma, has also been associated with fingolimod use, and a recent analysis noted a small increase in the overall risk of invasive cancer with fingolimod.^6^, ^46^ With respect to liver function, alanine and aspartate transaminase elevations are common across all four S1P receptor modulators.^6^, ^7^, ^8^, ^9^ For ozanimod and ponesimod, these elevations typically resolved within 2–4 weeks of continued treatment,^7^, ^8^ while for siponimod and fingolimod, these elevations returned to normal 1–2 months after treatment discontinuation.^6^, ^9^

Pulmonary effects were observed in the first 1–3 months of treatment with all S1P receptor modulators, with dose‐dependent reductions in forced expiratory volume.^6^, ^7^, ^8^, ^9^ Routine monitoring with pulmonary function tests is not recommended for any S1P receptor modulator. Spirometry and diffusion lung capacity of carbon monoxide evaluation should be performed when clinically indicated.

No significant new safety concerns were reported in long‐term studies, including for over 14 years for fingolimod, up to 8 years of continuous ozanimod exposure, 8 years for ponesimod, and >5 years for siponimod.^34^, ^35^, ^36^, ^37^ AEs most commonly reported with the four S1P receptor modulators in long‐term follow‐up studies were viral upper respiratory tract infection, headache, hypertension, lymphopenia, and nasopharyngitis.^34^, ^35^, ^36^, ^37^ However, instances of disease rebound were reported following discontinuation of fingolimod.^6^, ^47^ In most of these cases, patients did not return to the functional status they had before stopping fingolimod treatment.^6^ Symptoms generally occurred within 12 weeks but were reported for up to 24 weeks after discontinuation.^6^ One such condition, relapse with tumefactive demyelinating lesions, was observed within the initial 9 months following treatment initiation as well as 6 months after treatment discontinuation.^6^, ^48^ Due to the association of fingolimod with tumefactive MS, the condition should be considered when a severe MS relapse occurs during fingolimod treatment, especially during initiation, or after discontinuation of treatment, prompting imaging evaluation and subsequent appropriate treatment initiation.^6^, ^48^ To our knowledge, no cases of severe disease rebound were reported following the discontinuation of ozanimod, ponesimod, or siponimod.^8^, ^9^, ^49^ Mild to moderate relapses occurred in 2.3% of patients with RMS following ozanimod discontinuation, and 70% of those experienced a complete recovery within 30 days of onset.^50^

### Safety and tolerability during pregnancy

Animal studies demonstrated the potential for fetal harm with S1P receptor modulators in the absence of maternal toxicity.^6^, ^7^, ^8^, ^9^ S1P receptor modulators and/or their metabolites were also detected in the milk of treated lactating rats.^51^ Dedicated human pregnancy studies were not conducted.

Pooled data from clinical trials of fingolimod reported 66 pregnancies with in utero exposure (the total number of pregnancies including exposure to fingolimod, placebo, or IFN β‐1a, was 89).^52^ Among these, there were 28 live births, 9 spontaneous abortions, 24 elective abortions, and 5 that were ongoing or lost to follow up at the time.^52^ In the postmarketing fingolimod pregnancy registry, 6.0% of reported pregnancies resulted in major congenital malformations in live births; 6.6% in major congenital malformations in live births, stillbirths, and pregnancies that were terminated due to fetal anomalies.^53^ In the general population, the incidence of congenital malformations in live births is around 3% and the incidence of spontaneous abortion is estimated to range from 12% to 20%.^54^, ^55^, ^56^ The ozanimod trials reported 78 pregnancies, including 42 live births, 1 report of duplex kidney, 12 spontaneous abortions, and 15 elective abortions.^57^ Over the course of the ponesimod clinical program (which included the 30‐day period following treatment discontinuation), a total of 19 pregnancies were reported.^58^ Among these, six resulted in live, normal births; three were spontaneous abortions, and there were eight elective abortions. Of the 12 abortion cases, one reported benign hydatidiform mole, while fetal abnormalities were not known/reported for the other cases.^59^ As of March 2022, 31 pregnancies were reported in women treated with siponimod, with no events of congenital malformation or fetal/maternal complications.^60^

### Safety outcomes with concomitant SARS‐CoV‐2 infection

Considering the reduction in lymphocyte count observed with S1P receptor modulator treatment, it was initially thought that patients with MS undergoing treatment may be more susceptible to poor outcomes from COVID‐19.^61^ However, severe disease and mortality in patients treated with all four S1P receptor modulators were comparable to the general population.^35^, ^62^, ^63^

Some studies demonstrated that patients with MS treated with S1P receptor modulators, particularly fingolimod, may have limited ability to develop a humoral immune response after COVID‐19 vaccination.^64^, ^65^, ^66^, ^67^, ^68^ Fingolimod‐, ponesimod‐, or siponimod‐treated patients were seropositive after second and third mRNA vaccinations, but with lower immunoglobulin G levels compared with untreated patients.^68^ Following a second mRNA vaccine, 25% of fingolimod patients had a T‐cell response. Nearly all vaccinated participants with serological data in the ozanimod DAYBREAK open‐label extension study mounted a serologic response after full COVID‐19 vaccination.^69^ A literature review found that fingolimod treatment was associated with blunted antibody vaccine responses (seroconversion rate of 28%–77%) compared with untreated individuals, while seroconversion rates seen in the majority of people treated with siponimod, ozanimod, and ponesimod are higher (71%–100%).^63^ Differences in (COVID‐19) vaccine responses between fingolimod and newer S1P receptor modulators may be due to differences in S1P receptor subtype modulation.^63^ Fingolimod targets S1P_1_, S1P_3_, S1P_4_, and S1P_5_, while ponesimod, siponimod, and ozanimod target S1P_1_, and siponimod and ozanimod also target S1P_5_.^63^ The stronger antibody vaccine responses of ponesimod, siponimod, and ozanimod may be due to their limited impact on S1P_3_ and S1P_4_ and their effect on the formation and function of the germinal center, important in generating neoantigen antibody responses.^63^ While, reduced T‐cell responses have been reported with fingolimod, data on the peripheral blood T‐cell responses in patients treated with second generation S1P modulators have been inconsistent.^63^

## Clinical considerations when prescribing S1P receptor modulators to patients with multiple sclerosis

Clinical considerations for patients with MS who are candidates for S1P receptor modulator therapy are listed in Table 4.

**Table 4:** Clinical considerations before initiating treatment, at first dose, and throughout treatment.

	Fingolimod^6^ S1P_1_, S1P_3_, S1P_4_, and S1P_5_	Ozanimod^7^ S1P_1_ and S1P_5_	Ponesimod^8^ S1P_1_	Siponimod^9^ S1P_1_ and S1P_5_
Lymphocyte dynamics				
Maximum lymphocyte reduction from baseline	70%–80%	55%	60%–70%	70%–80%
Time to lymphocyte restoration after discontinuation	4–8 weeks	Median of 30 days with 90% in normal range within 3 months	Within 1 week for 90% of patients	10 days for most patients (90%) but can take up to 3–4 weeks
Pretreatment assessments				
Genetic	NA	NA	NA	Genotype for CYP2C9
Cardiac	ECG	ECG	ECG	ECG
Hepatic	Transaminase and bilirubin from the past 6 months	Transaminase and bilirubin from the past 6 months	Transaminase and bilirubin from the past 6 months	Transaminase and bilirubin from the past 6 months
Blood	Recent^a^ CBC	Recent^a^ CBC	Recent^a^ CBC	Recent^a^ CBC
Antibodies	Immunity to VZV HPV vaccination and pap test	Antibodies to VZV or VZV vaccination	Antibodies to VZV or VZV vaccination	Antibodies to VZV or VZV vaccination
Ophthalmic examinations	All patients	At‐risk patients^b^	All patients	All patients
Skin examinations	Periodic skin examination is recommended for all patients, particularly those with risk factors for skin cancer	NA	NA	All patients
First‐dose monitoring				
Requires dose titration	No	Yes, 8 days	Yes, 15 days	Yes, 5 days
Requires first‐dose monitoring	Yes, 6 h for all patients Overnight for those with bradycardia or other cardiovascular conditions	No	Recommended for 4 h for patients with bradycardia, first‐ or second‐degree (Mobitz type I) AV block, or a history of myocardial infarction or heart failure	Recommended for 6 h for patients with bradycardia, first‐ or second‐degree (Mobitz type I) AV block, or a history of myocardial infarction or heart failure
Continued monitoring during treatment				
Blood pressure	Monitor and manage appropriately	Monitor and manage appropriately	Monitor and manage appropriately	Monitor and manage appropriately
Ocular examinations	Regular examinations of the fundus and macula for patients with a history of uveitis or diabetes mellitus, and for all patients whenever a change in vision is noted	Regular examinations of the fundus and macula for patients with a history of uveitis or diabetes mellitus, and for all patients whenever a change in vision is noted	Regular examinations of the fundus and macula for patients with a history of uveitis or diabetes mellitus, and for all patients whenever a change in vision is noted	Regular examinations of the fundus and macula for patients with a history of uveitis or diabetes mellitus, and for all patients whenever a change in vision is noted
Serious infections	Monitor during treatment and during the time of lymphocyte restoration after discontinuation	Monitor during treatment and during the time of lymphocyte restoration after discontinuation	Monitor during treatment and during the time of lymphocyte restoration after discontinuation	Monitor during treatment and during the time of lymphocyte restoration after discontinuation
Skin examinations	Examine annually by a dermatologist; evaluate any suspicious skin lesions	–	Examine annually by a dermatologist; evaluate any suspicious skin lesions	Examine‐ annually by a dermatologist; evaluate any suspicious skin lesions
Spirometric evaluations	If clinically indicated	If clinically indicated	If clinically indicated	If clinically indicated
Complete blood count	Monitor during treatment	Monitor during treatment	Monitor during treatment	Monitor during treatment
Liver aminotransferase levels	Checked periodically	Checked if clinically indicated	Checked if clinically indicated	Checked if clinically indicated

AV, atrioventricular; BP, blood pressure; bpm, beats per minute; CBC, complete blood count; CYP2C9, cytochrome P450 family 2 subfamily C member 9; ECG, electrocardiogram; HPV, human papillomavirus; HR, heart rate; MRI, magnetic resonance imaging; MS, multiple sclerosis; QTc, corrected QT interval; S1P_1_, sphingosine 1‐phosphate receptor subtype 1; S1P_3_, sphingosine 1‐phosphate receptor subtype 3; S1P_4_, sphingosine 1‐phosphate receptor subtype 4; S1P_5_, sphingosine 1‐phosphate receptor subtype 5; VZV, varicella‐zoster virus.

^a^ Within 6 months or after discontinuation of prior MS therapy.

^b^ Ophthalmic evaluation is recommended for patients with diabetes mellitus or uveitis, prior to treatment initiation with follow‐up.

### Contraindications

Certain contraindications are unique to individual agents. For example, fingolimod is contraindicated in patients with a baseline QTc interval ≥ 500 msec and cardiac arrhythmias requiring anti‐arrhythmic treatment with Class Ia or Class III anti‐arrhythmic drugs^6^; ozanimod in patients with sino‐atrial block, severe untreated sleep apnea, and those taking an MAO inhibitor^7^; ponesimod in patients with sino‐atrial block^8^; and siponimod in patients with CYP2C9*3/*3 genotype.^9^ Contraindications for all four S1P receptor modulators are listed in Table 1.

#### Additional considerations for specific drug–drug interactions

Live, attenuated vaccines should be avoided during treatment with all four S1P receptor modulators and for 4 weeks following discontinuation.^6^, ^7^, ^8^, ^9^ In addition, clinicians are advised to use caution when concomitantly prescribing beta‐blockers, digoxin, calcium‐channel blockers, or QT‐prolonging therapies, considering the cardiovascular effects of S1P receptor modulators. Antineoplastic, immune‐modulating, or corticosteroid or non‐corticosteroid immunosuppressive therapies should also be used with caution owing to additive effects with S1P receptor modulators.

Some clinical considerations apply only to a particular medication in this class, owing to specific interactions. Coadministration of ozanimod with opioids, selective serotonin reuptake inhibitors (SSRI), serotonin‐norepinephrine reuptake inhibitors (SNRI), tricyclic antidepressants, or tyramine is not recommended.^7^ Although a post hoc analysis of the DAYBREAK study did not find an excess number of adverse events, including serotonin syndrome–related TEAEs or hypertension in patients taking an SSRI or SNRI in combination with ozanimod.^70^ Concomitant use of beta‐blockers and nondihydropyridine calcium channel blockers with ponesimod could lead to severe bradycardia and heart block; therefore, advice from a cardiologist should be sought before treatment initiation to determine the most appropriate monitoring for patients prescribed these medications.^8^ Administration of ponesimod is also not recommended with strong CYP3A4 and UGT1A1 inducers (e.g., rifampin, phenytoin, carbamazepine).^8^ Concomitant use of siponimod with modafinil plus all CYP2C9 and CYP3A4 inhibitors or inducers is not recommended due to a significant increase or decrease in siponimod exposure, respectively.^9^

### Pretreatment assessments

Patients initiating treatment with siponimod should be tested to determine CYP2C9 genotype.^9^ Considering the cardiovascular effects of S1P receptor modulators, an electrocardiogram should be obtained to determine whether preexisting conduction abnormalities are present.^6^, ^7^, ^8^, ^9^ A cardiologist should be consulted before initiating treatment in patients with significant QT prolongation, arrhythmias requiring treatment, ischemic heart disease, heart failure, history of cardiac arrest or myocardial infarction, cerebrovascular disease, uncontrolled hypertension, second‐degree Mobitz type II or higher AV block, sick‐sinus syndrome, or sinoatrial heart block.^6^, ^7^, ^8^, ^9^ Transaminase and bilirubin levels and a complete blood count should be obtained before initiating S1P receptor modulators.^6^, ^7^, ^8^, ^9^ Patients without a confirmed history of chickenpox or without documentation of a full course of vaccination against varicella zoster virus (VZV) should be tested for antibodies to VZV before initiating S1P receptor modulators^6^, ^7^, ^8^, ^9^; VZV vaccination of antibody‐negative patients is recommended before starting treatment with ozanimod, ponesimod, fingolimod, and siponimod.^6^, ^7^, ^8^, ^9^ An ophthalmologic evaluation should be performed by an ophthalmologist for all patients starting fingolimod, ponesimod, and siponimod, and at‐risk patients starting ozanimod.^6^, ^7^, ^8^, ^9^ The ophthalmologic evaluation should be conducted throughout the treatment period of the S1P receptor modulator in patients with a history of uveitis, macular edema, and diabetes mellitus.^71^ A skin examination should be performed by a dermatologist in all patients before starting siponimod.^9^

### First dose monitoring and dose escalation protocols

When treating patients with S1P receptor modulators, the most important clinical consideration is the effect of initial dosing on heart rate and subsequent hypertension.^72^ Clinicians should follow the recommended guidelines for initial titration of ozanimod until day 7, ponesimod until day 14, and siponimod until day 4 and day 5 to reach the 1 and 2 mg maintenance dose, respectively.^7^, ^8^, ^9^ First‐dose monitoring is recommended depending on the specific agent and comorbidities in a setting where resources are available to appropriately manage symptomatic bradycardia as described in Table 4 for fingolimod, ponesimod, and siponimod.^6^, ^8^, ^9^ First‐dose monitoring is not noted as required in the label for ozanimod. In patients with mild or moderate hepatic impairment (Child‐Pugh class A or B) beginning ozanimod, the prescribing information recommends an every‐other‐day dosing schedule following the 7‐day titration period^7^; however, there is a lack of published data for this recommendation.

### Continued monitoring during treatment

Continued and regular monitoring of patients during treatment should be considered based on the AE profile of the S1P receptor modulator chosen for therapy.^6^, ^7^, ^8^, ^9^ Monitoring should focus on blood pressure, ocular examinations, possible serious infections, skin examinations, white blood cell and lymphocyte counts, and liver aminotransferase levels (Table 4). Skin examinations and screening for cutaneous malignancies should be performed annually by a dermatologist in people receiving treatment when indicated. Clinicians should not only monitor patients for serious infections during treatment but also continue monitoring after discontinuation of therapy, until lymphocyte levels are restored.^6^, ^7^, ^8^, ^9^ Patients should be counseled about the risk for progressive multifocal leukoencephalopathy (PML). If PML is suspected, based on symptomatic and/or magnetic resonance imaging findings, treatment with S1P receptor modulators should be discontinued.^6^, ^7^, ^8^, ^9^ PML is a rare infection that predominantly occurs in cases of prolonged moderate‐to‐severe lymphopenia, and in older patients.^73^ So far, in clinical studies, PML occurred in 61 patients treated with fingolimod,^74^ three patients treated with siponimod,^75^ one patient treated with ozanimod,^35^ and no patients treated with ponesimod.^8^ These data indicate that there is a risk of PML in fingolimod‐treated patients, particularly in those of older age. Immune reconstitution inflammatory syndrome (IRIS) has also been reported in MS patients who developed PML, generally within a few months of S1P receptor modulator discontinuation. Patients who discontinue treatment due to PML should be monitored for the development of IRIS.^7^ A small number of cases of posterior reversible encephalopathy syndrome (PRES) have occurred with fingolimod use. Symptoms include sudden onset severe headache, visual disturbances, altered mental status, and seizure. If PRES is not promptly treated, it can lead to ischemic stroke or cerebral hemorrhage. Treatment should be discontinued if PRES is suspected.^6^ Clinicians should educate patients of childbearing potential of the requirement for effective contraception during treatment and for the prescribed duration following treatment discontinuation. Concomitant use of S1P receptor modulators is not expected to decrease the efficacy of hormonal contraceptives based on a lack of relevant pharmacokinetic interaction between these medications. This included the coadministration of fingolimod 0.5 mg daily or ozanimod with an oral contraceptive containing ethinyl estradiol and norethindrone, coadministration of ponesimod with an oral hormonal contraceptive containing 1 mg norethisterone/norethindrone and 35 μg ethinyl estradiol, and coadministration of siponimod with an oral contraceptive containing 30 μg ethinyl estradiol and 150 μg levonorgestrel.^6^, ^7^, ^8^, ^9^ All patients receiving S1P receptor modulators should be made aware of the risk of relapse causing severe disability following treatment discontinuation, particularly those treated with fingolimod. If this occurs, it is typically within 3 months of treatment cessation and may be associated with IRIS. Thus far, there is no evidence of rebound secondary to discontinuation when using the newer S1P receptor modulators. Importantly, a clear‐cut plan to monitor and treat exacerbations should be in place.

## Conclusions

S1P receptor modulators have emerged as an oral disease‐modifying treatment option for MS that has a unique mechanism of action compared with other therapies. While efficacy data across the pivotal clinical trials of fingolimod, ozanimod, ponesimod, and siponimod appear to be relatively similar in patients with MS, each agent is unique and may be better suited for specific patients. For example, siponimod was the first S1P receptor modulator to be approved for patients with active SPMS; although the other agents could potentially be effective in this disease state, their efficacy in SPMS has not been proven. Clarification is needed whether all S1P receptor modulators have rebound effects, and more data are needed regarding their impact on vaccine response and the risk for infections, including PML.

## Author Contributions

All authors have contributed to the writing of the manuscript and have seen and approved the submitted version.

## Funding Information

This review was supported by Bristol Myers Squibb, Princeton, NJ.

## Conflict of Interest

**PKC** has consulted for Accordant, Biogen, Bristol Myers Squibb, Eli Lilly and Company, Genentech, GlaxoSmithKline, Horizon Therapeutics, LabCorp, Mylan, Novartis, Sanofi/Genzyme, and TG Therapeutics; and has received research support from Actelion, Alkermes, Celgene, CorEvitas, Genentech/Roche, Janssen Pharmaceuticals, MedDay, NINDS, Novartis, and Sanofi/Genzyme. **MSF** has received research or educational grants from Sanofi/Genzyme Canada; received honoraria or consultation fees from Alexion/Astra Zeneca, Biogen Idec, EMD Inc/EMD Serono/Merck Serono, F. Hoffmann‐La Roche, Find Therapeutics, Novartis, Quanterix, Sanofi/Genzyme, and Teva Canada Innovation; is a member of a company advisory board, board of directors or other similar group of Actelion/Janssen (J&J), Alexion/Astra Zeneca, Atara Biotherapeutics, Bayer Healthcare, Celestra Health, EMD Inc./Merck Serono, F. Hoffmann‐La Roche, Find Therapeutics, Novartis, Sanofi/Genzyme, and Setpoint Medical; has participated in a company sponsored speaker's bureau for Sanofi/Genzyme and EMD Serono. **BAC** has received personal compensation from Mylan for consulting, and from Applied Clinical Intelligence for serving as an external adjudicator for a clinical trial conducted by Sanofi. **BACC** reports personal compensation for consulting for Alexion, Atara, Autobahn, Avotres, Biogen, Boston Pharma, EMD Serono, Gossamer Bio, Hexal/Sandoz, Horizon, Immunic AG, Kyverna, Neuron23, Novartis, Sanofi, and TG Therapeutics and research support from Genentech. **CEM** has consulted for Alexion, ANI, Banner Life Sciences, Biogen, Bristol Myers Squibb, EMD Serono, Genentech/Roche, Sanofi/Genzyme, Horizon Therapeutics, Immunic AG, Janssen/Actelion, Novartis, and TG Therapeutics.

## Supporting information


Figure S1.

## References

[1] McGinley MP , Cohen JA . Sphingosine 1‐phosphate receptor modulators in multiple sclerosis and other conditions. Lancet. 2021;398:1184‐1194. 34175020 10.1016/S0140-6736(21)00244-0

[2] Chun J , Giovannoni G , Hunter SF . Sphingosine 1‐phosphate receptor modulator therapy for multiple sclerosis: differential downstream receptor signalling and clinical profile effects. Drugs. 2021;81:207‐231. 33289881 10.1007/s40265-020-01431-8 PMC7932974

[3] Zhao Z , Lv Y , Gu ZC , Ma CL , Zhong MK . Risk for cardiovascular adverse events associated with sphingosine‐1‐phosphate receptor modulators in patients with multiple sclerosis: insights from a pooled analysis of 15 randomised controlled trials. Front Immunol. 2021;12:795574. 34950154 10.3389/fimmu.2021.795574 PMC8688957

[4] Forrest M , Sun SY , Hajdu R , et al. Immune cell regulation and cardiovascular effects of sphingosine 1‐phosphate receptor agonists in rodents are mediated via distinct receptor subtypes. J Pharmacol Exp Ther. 2004;309:758‐768. 14747617 10.1124/jpet.103.062828

[5] Musella A , Gentile A , Guadalupi L , et al. Central modulation of selective sphingosine‐1‐phosphate receptor 1 ameliorates experimental multiple sclerosis. Cell. 2020;9:1290. 10.3390/cells9051290 PMC7291065 32455907

[6] Gilenya [package insert]. Novartis; 2023.

[7] Zeposia [package insert]. Bristol Myers Squibb; 2023.

[8] Ponvory [package insert]. Janssen Pharmaceuticals, Inc.; 2022.

[9] Mayzent [package insert]. Novartis Pharmaceuticals Corporation; 2023.

[10] Gilenya [summary of product characteristics]. Novartis Europharm Limited; 2022.

[11] Mayzent [summary of product characteristics]. Novartis Europharm Limited; 2022.

[12] Zeposia [summary of product characteristics]. Celgene Distribution B.V.; 2023.

[13] Ponvory [summary of product characteristics]. Janssen‐Cilag International NV; 2022.

[14] Pérez‐Jeldres T , Alvarez‐Lobos M , Rivera‐Nieves J . Targeting sphingosine‐1‐phosphate signaling in immune‐mediated diseases: beyond multiple sclerosis. Drugs. 2021;81:985‐1002. 33983615 10.1007/s40265-021-01528-8 PMC8116828

[15] Bravo G , Cedeño RR , Casadevall MP , et al. Sphingosine‐1‐phosphate (S1P) and S1P signaling pathway modulators, from current insights to future perspectives. Cell. 2022;11:2058. 10.3390/cells11132058 PMC9265592 35805142

[16] Lorscheider J , Benkert P , Lienert C , et al. Comparative analysis of dimethyl fumarate and fingolimod in relapsing‐remitting multiple sclerosis. J Neurol. 2021;268:941‐949. 32974794 10.1007/s00415-020-10226-6 PMC7914245

[17] Roy R , Alotaibi AA , Freedman MS . Sphingosine 1‐phosphate receptor modulators for multiple sclerosis. CNS Drugs. 2021;35:385‐402. 33797705 10.1007/s40263-021-00798-w

[18] Tascenso ODT [package insert]. Handa Neuroscience; 2022.

[19] Tran JQ , Zhang P , Ghosh A , et al. Single‐dose pharmacokinetics of ozanimod and its major active metabolites alone and in combination with gemfibrozil, itraconazole, or rifampin in healthy subjects: a randomized, parallel‐group, open‐label study. Adv Ther. 2020;37:4381‐4395. 32857315 10.1007/s12325-020-01473-0

[20] Choi D , Stewart AP , Bhat S . Ozanimod: a first‐in‐class sphingosine 1‐phosphate receptor modulator for the treatment of ulcerative colitis. Ann Pharmacother. 2021;56:592‐599. 34423657 10.1177/10600280211041907

[21] Correale J , Halfon MJ , Jack D , Rubstein A , Villa A . Acting centrally or peripherally: a renewed interest in the central nervous system penetration of disease‐modifying drugs in multiple sclerosis. Mult Scler Relat Disord. 2021;56:103264. 34547609 10.1016/j.msard.2021.103264

[22] Hartung HP , Cree BAC , Barnett M , Meuth SG , Bar‐Or A , Steinman L . Bioavailable central nervous system disease‐modifying therapies for multiple sclerosis. Front Immunol. 2023;14:1290666. 38162670 10.3389/fimmu.2023.1290666 PMC10755740

[23] Favaretto A , Lazzarotto A , Margoni M , Poggiali D , Gallo P . Effects of disease modifying therapies on brain and grey matter atrophy in relapsing remitting multiple sclerosis. Mult Scler Demyelinating Disord. 2018;3:1.

[24] Skeen MB . Sphingosine‐1‐phosphate modulators for multiple sclerosis. Pract Neurol. 2020;28‐32. https://practicalneurology.com/articles/2020‐feb/sphingosine‐1‐phosphate‐modulators‐for‐multiple‐sclerosis/pdf

[25] Kappos L , Radue EW , O'Connor P , et al. A placebo‐controlled trial of oral fingolimod in relapsing multiple sclerosis. N Engl J Med. 2010;362:387‐401. 20089952 10.1056/NEJMoa0909494

[26] Comi G , Kappos L , Selmaj KW , et al. Safety and efficacy of ozanimod versus interferon beta‐1a in relapsing multiple sclerosis (SUNBEAM): a multicentre, randomised, minimum 12‐month, phase 3 trial. Lancet Neurol. 2019;18:1009‐1020. 31492651 10.1016/S1474-4422(19)30239-X

[27] Cohen JA , Comi G , Selmaj KW , et al. Safety and efficacy of ozanimod versus interferon beta‐1a in relapsing multiple sclerosis (RADIANCE): a multicentre, randomised, 24‐month, phase 3 trial. Lancet Neurol. 2019;18:1021‐1033. 31492652 10.1016/S1474-4422(19)30238-8

[28] Kappos L , Fox RJ , Burcklen M , et al. Ponesimod compared with teriflunomide in patients with relapsing multiple sclerosis in the active‐comparator phase 3 OPTIMUM study: a randomized clinical trial. JAMA Neurol. 2021;78:558‐567. 33779698 10.1001/jamaneurol.2021.0405 PMC8008435

[29] Kappos L , Bar‐Or A , Cree BAC , et al. Siponimod versus placebo in secondary progressive multiple sclerosis (EXPAND): a double‐blind, randomised, phase 3 study. Lancet. 2018;391:1263‐1273. 29576505 10.1016/S0140-6736(18)30475-6

[30] Calabresi PA , Radue EW , Goodin D , et al. Safety and efficacy of fingolimod in patients with relapsing‐remitting multiple sclerosis (FREEDOMS II): a double‐blind, randomised, placebo‐controlled, phase 3 trial. Lancet Neurol. 2014;13:545‐556. 24685276 10.1016/S1474-4422(14)70049-3

[31] Cohen JA , Barkhof F , Comi G , et al. Oral fingolimod or intramuscular interferon for relapsing multiple sclerosis. N Engl J Med. 2010;362:402‐415. 20089954 10.1056/NEJMoa0907839

[32] Chitnis T , Arnold DL , Banwell B , et al. Trial of fingolimod versus interferon beta‐1a in pediatric multiple sclerosis. N Engl J Med. 2018;379:1017‐1027. 30207920 10.1056/NEJMoa1800149

[33] Lublin F , Miller DH , Freedman MS , et al. Oral fingolimod in primary progressive multiple sclerosis (INFORMS): a phase 3, randomised, double‐blind, placebo‐controlled trial. Lancet. 2016;387:1075‐1084. 26827074 10.1016/S0140-6736(15)01314-8

[34] Cohen JA , Tenenbaum N , Bhatt A , et al. Extended treatment with fingolimod for relapsing multiple sclerosis: the 14‐year LONGTERMS study results. Ther Adv Neurol Disord. 2019;12:1756286419878324. doi:10.1177/1756286419878324 31598139 PMC6763939

[35] Cree BA , Selmaj KW , Steinman L , et al. Long‐term safety and efficacy of ozanimod in relapsing multiple sclerosis: up to 5 years of follow‐up in the DAYBREAK open‐label extension trial. Mult Scler. 2022;28:1944‐1962. 35765217 10.1177/13524585221102584 PMC9493410

[36] Freedman MS , Pozzilli C , Havrdova EK , et al. Long‐term treatment with ponesimod in relapsing‐remitting multiple sclerosis: results from randomized phase 2b core and extension studies. Neurology. 2022;99:e762‐e774. 35667837 10.1212/WNL.0000000000200606 PMC9484728

[37] Cree BA , Arnold DL , Fox RJ , et al. Long‐term efficacy and safety of siponimod in patients with secondary progressive multiple sclerosis: analysis of EXPAND core and extension data up to >5 years. Mult Scler. 2022;28:1591‐1605. 35380078 10.1177/13524585221083194 PMC9315196

[38] Choden T , Cohen NA , Rubin DT . Sphingosine‐1 phosphate receptor modulators: the next wave of oral therapies in inflammatory bowel disease. Gastroenterol Hepatol. 2022;18:265‐271. PMC9666818 36397756

[39] Sharma K , Chaudhary D , Beard K , Srivastava S , Khalid SH , Sriwastava S . A comprehensive review of varicella‐zoster virus, herpes simplex virus and cryptococcal infections associated with sphingosine‐1‐phosphate receptor modulators in multiple sclerosis patients. Mult Scler Relat Disord. 2022;59:103675. 35168095 10.1016/j.msard.2022.103675

[40] Chaplin S . Ozanimod for treating relapsing‐remitting multiple sclerosis. Prescriber. 2021;32:32‐33.

[41] Piri Cinar B , Konuskan B , Anlar B , Ozakbas S . Narrative review based on fingolimod therapy in pediatric MS. SAGE Open Med. 2023;11:20503121231171996. 37181277 10.1177/20503121231171996 PMC10170592

[42] Comi G , Hartung HP , Bakshi R , Williams IM , Wiendl H . Benefit‐risk profile of sphingosine‐1‐phosphate receptor modulators in relapsing and secondary progressive multiple sclerosis. Drugs. 2017;77:1755‐1768. 28905255 10.1007/s40265-017-0814-1 PMC5661009

[43] Brown B , Weiss JL , Kolodny S , Meng X , Williams IM , Osborne JA . Analysis of cardiac monitoring and safety data in patients initiating fingolimod treatment in the home or in clinic. BMC Neurol. 2019;19:287. 31729968 10.1186/s12883-019-1506-0 PMC6857316

[44] Mandal P , Gupta A , Fusi‐Rubiano W , Keane PA , Yang Y . Fingolimod: therapeutic mechanisms and ocular adverse effects. Eye. 2017;31:232‐240. 27886183 10.1038/eye.2016.258 PMC5306460

[45] Tiper IV , East JE , Subrahmanyam PB , Webb TJ . Sphingosine 1‐phosphate signaling impacts lymphocyte migration, inflammation and infection. Pathog Dis. 2016;74:1‐9. 10.1093/femspd/ftw063 PMC5985508 27354294

[46] Alping P , Askling J , Burman J , et al. Cancer risk for fingolimod, natalizumab, and rituximab in MS patients. Ann Neurol. 2020;87:688‐699. 32056253 10.1002/ana.25701

[47] Hatcher SE , Waubant E , Nourbakhsh B , Crabtree‐Hartman E , Graves JS . Rebound syndrome in patients with multiple aclerosis after cessation of fingolimod treatment. JAMA Neurol. 2016;73:790‐794. 27135594 10.1001/jamaneurol.2016.0826

[48] Croteau D , Tobenkin A , Brinker A , Kortepeter CM . Tumefactive multiple sclerosis in association with fingolimod initiation and discontinuation. Mult Scler. 2021;27:903‐912. 32662718 10.1177/1352458520938354

[49] Selmaj KW , Cohen JA , Comi G , et al. Ozanimod in relapsing multiple sclerosis: pooled safety results from the clinical development program. Mult Scler Relat Disord. 2021;51:102844. 33892317 10.1016/j.msard.2021.102844

[50] Gold R , Selmaj KW , Berkovitch R , et al. Analysis of multiple sclerosis (MS) relapse following discontinuation of ozanimod in DAYBREAK [poster EPO‐131]. Presented at: Annual Congress of the European Academy of Neurology; June 25–28, 2022; Vienna, Austria.

[51] Simone IL , Tortorella C , Ghirelli A . Influence of pregnancy in multiple sclerosis and impact of disease‐modifying therapies. Front Neurol. 2021;12:697974. 34276545 10.3389/fneur.2021.697974 PMC8280312

[52] Karlsson G , Francis G , Koren G , et al. Pregnancy outcomes in the clinical development program of fingolimod in multiple sclerosis. Neurology. 2014;82:674‐680. 24463630 10.1212/WNL.0000000000000137 PMC3945658

[53] Reproductive toxicity. 2022. Accessed May 12, 2023. https://www.fingolimodinfo.com/en/reproductive‐toxicity

[54] Garcia‐Enguidanos A , Calle ME , Valero J , et al. Risk factors in miscarriage: a review. Eur J Obstet Gynecol Reprod Biol. 2002;102:111‐119. 11950476 10.1016/s0301-2115(01)00613-3

[55] Rossen LM , Ahrens KA , Branum AM . Trends in risk of pregnancy loss among US women, 1990‐2011. Paediatr Perinat Epidemiol. 2018;32:19‐29. 29053188 10.1111/ppe.12417 PMC5771868

[56] Data & Statistics on Birth Defects . 2023. Accessed July 5, 2023. https://www.cdc.gov/ncbddd/birthdefects/data.html

[57] Krakovich A , Comi G , Dubinsky MC , et al. Pregnancy outcomes in the ozanimod clinical development program in relapsing multiple sclerosis, ulcerative colitis, and Crohn's disease [poster DMT17]. Presented at: Annual Meeting of the Consortium of Multiple Sclerosis Centers; May 31–June 3, 2023; Aurora, CO.

[58] Use of PONVORY in Pregnancy and in Females of Reproductive Potential . 2022. Accessed June 29, 2023. https://www.janssenscience.com/products/ponvory/medical‐content/use‐of‐ponvory‐in‐pregnancy‐and‐in‐females‐of‐reproductive‐potential

[59] Rosas‐Ballina M , Wooller A , Jones R , et al. Fetal exposure with ponesimod treatment across clinical development studies [abstract P080]. Mult Scler. 2022;28(1 suppl):56.

[60] Siponimod Key Safety Topics: Reproductive toxicity . 2023. Accessed June 30, 2023. https://www.siponimodinfo.com/en/key‐safety‐topics/reproductive‐toxicity

[61] Huang I , Pranata R . Lymphopenia in severe coronavirus disease‐2019 (COVID‐19): systematic review and meta‐analysis. J Intensive Care. 2020;8:36. 32483488 10.1186/s40560-020-00453-4 PMC7245646

[62] Sullivan R , Kilaru A , Hemmer B , et al. COVID‐19 infection in fingolimod‐ or siponimod‐treated patients: case series. Neurol Neuroimmunol Neuroinflamm. 2022;9:e1092. 10.1212/NXI.0000000000001092 PMC8860466 34848501

[63] Baker D , Forte E , Pryce G , et al. The impact of sphinogosine‐1‐phosphate receptor modulators on COVID‐19 and SARS‐CoV‐2 vaccination. Mult Scler Relat Disord. 2023;69:104425. 36470168 10.1016/j.msard.2022.104425 PMC9678390

[64] Achiron A , Mandel M , Dreyer‐Alster S , et al. Humoral immune response in multiple sclerosis patients following PfizerBNT162b2 COVID19 vaccination: up to 6 months cross‐sectional study. J Neuroimmunol. 2021;361:577746. 34655991 10.1016/j.jneuroim.2021.577746 PMC8500842

[65] Achiron A , Mandel M , Gurevich M , et al. Immune response to the third COVID‐19 vaccine dose is related to lymphocyte count in multiple sclerosis patients treated with fingolimod. J Neurol. 2022;269:2286‐2292. 35235002 10.1007/s00415-022-11030-0 PMC8889521

[66] Bsteh G , Dürauer S , Assar H , et al. Humoral immune response after COVID‐19 in multiple sclerosis: a nation‐wide Austrian study. Mult Scler. 2021;27:2209‐2218. 34595968 10.1177/13524585211049391 PMC8597187

[67] Jakimovski D , Zakalik K , Awan S , et al. COVID‐19 vaccination in multiple sclerosis and inflammatory diseases: effects from disease‐modifying therapy, long‐term seroprevalence and breakthrough infections. Vaccine. 2022;10:695. 10.3390/vaccines10050695 PMC9146267 35632451

[68] Milo R , Staun‐Ram E , Karussis D , et al. Humoral and cellular immune responses to SARS‐CoV‐2 mRNA vaccination in patients with multiple sclerosis: an Israeli multi‐center experience following 3 vaccine doses. Front Immunol. 2022;13:868915. 35432335 10.3389/fimmu.2022.868915 PMC9012137

[69] Cree BA , Maddux R , Bar‐Or A , et al. Serologic response and clinical outcomes of SARS‐CoV‐2 infection and vaccination in ozanimod‐treated participants with relapsing multiple sclerosis [abstract P1199]. Mult Scler J. 2022;28(suppl 3):973‐974.

[70] Naismith RT , Cohen JT , Bar‐Or A , et al. Safety of concurrent administration of ozanimod and serotonergic antidepressants in patients with relapsing multiple sclerosis [poster P080]. Presented at: Annual Forum of the Americas Committee for Treatment and Research in Multiple Sclerosis; February 25–27, 2021.

[71] Dumitrescu L , Papathanasiou A , Coclitu C , et al. An update on the use of sphingosine 1‐phosphate receptor modulators for the treatment of relapsing multiple sclerosis. Expert Opin Pharmacother. 2023;24:495‐509. 36946625 10.1080/14656566.2023.2178898 PMC10069376

[72] Constantinescu V , Haase R , Akgün K , Ziemssen T . S1P receptor modulators and the cardiovascular autonomic nervous system in multiple sclerosis: a narrative review. Ther Adv Neurol Disord. 2022;15:17562864221133163. 36437849 10.1177/17562864221133163 PMC9685213

[73] Smith TE , Kister I . Infection mitigation strategies for multiple sclerosis patients on Oral and monoclonal disease‐modifying therapies. Curr Neurol Neurosci Rep. 2021;21:36. 34009478 10.1007/s11910-021-01117-y PMC8132488

[74] Progressive multifocal leukoencephalopathy: key safety topics. 2022. Accessed August 17, 2023. https://www.fingolimodinfo.com/en/progressive‐multifocal‐leukoencephalopathy‐0

[75] Progressive multifocal leukoencephalopathy: key safety topics. 2023. Accessed October 17, 2023. https://www.siponimodinfo.com/en‐us/key‐safety‐topics/pml

