# Supplementary material for: Sphingosine 1‐phosphate receptor modulators in multiple sclerosis treatment: A practical review
Source: Ann Clin Transl Neurol. 2024 Feb 16;11(4):842–55. doi: 10.1002/acn3.52017 (PMC11021614; doi:10.1002/acn3.52017)
Supplement: Supplementary file 1 — Figure S1. [file ACN3-11-842-s001.docx]

**Supplement to Sphingosine 1-phosphate receptor modulators for the treatment of multiple sclerosis: a practical review**

Patricia K. Coyle, Mark S. Freedman, Bruce A. Cohen, Bruce A.C. Cree, Clyde E. Markowitz

**Supplemental Figure 1.** Overview of S1P receptor subtypes and receptor selectivity of S1P receptor modulators.^1,2^

**
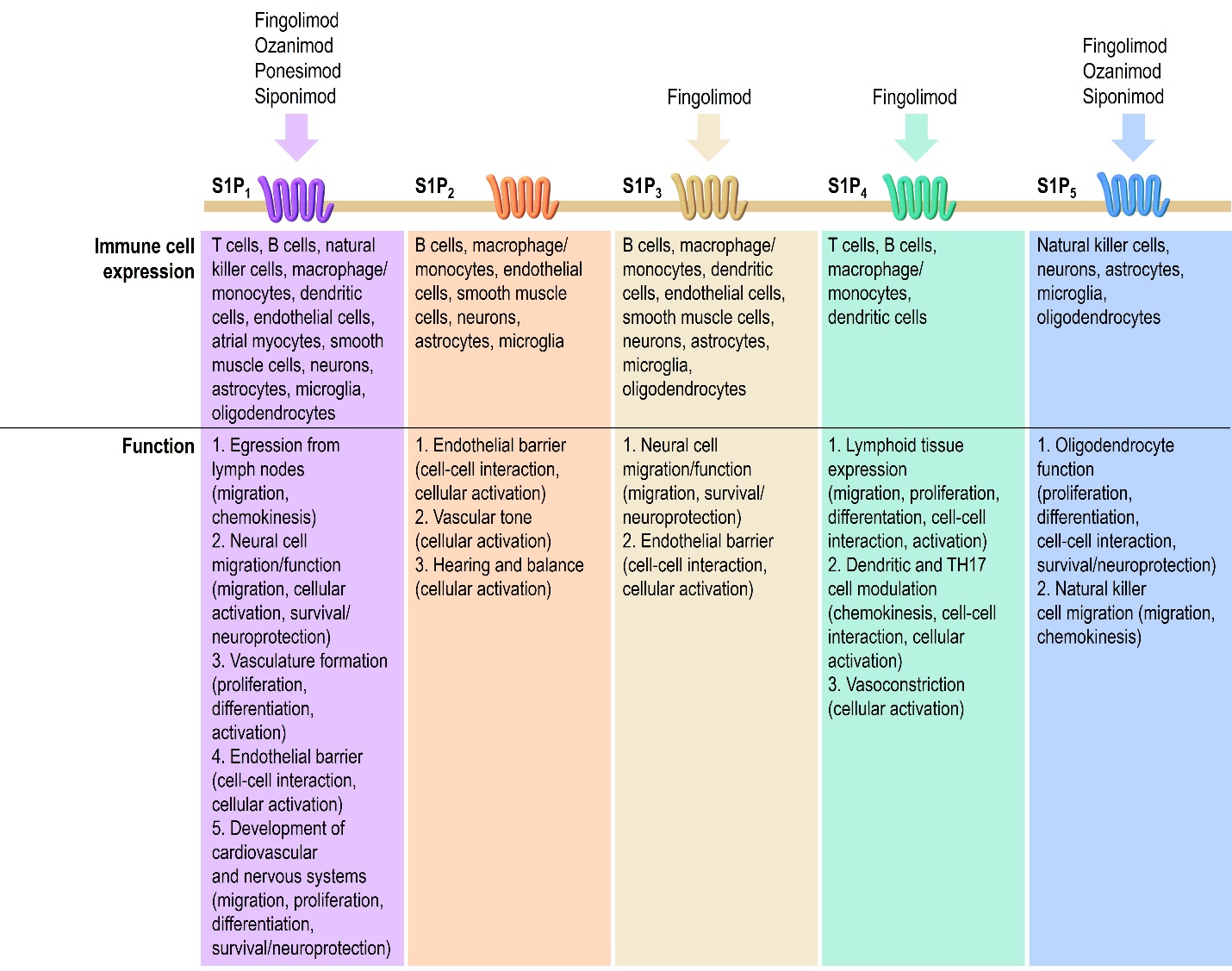
**

**References**

1. Chun J, Giovannoni G, Hunter SF. Sphingosine 1-phosphate receptor modulator therapy for multiple sclerosis: differential downstream receptor signalling and clinical profile effects. Drugs 2021;81:207-231.

2. Comi G, Hartung H-P, Bakshi R, Williams IM, Wiendl H. Benefit-risk profile of sphingosine-1-phosphate receptor modulators in relapsing and secondary progressive multiple sclerosis. Drugs 2017;77:1755-1768.
